# Annotations

**Published:** 1910-07-30

**Authors:** 


### ANNOTATIONS.

### The International Committee for Graduate Study in Medicine.

THE International Committee for Graduate Study in Medicine has decided to hold its first general meeting on October 9 next, at half-past twelve in the afternoon, at Berlin, in the historic Kaiserin Friedrich Haus on the Luisenplatz. The agenda deals with various questions of organisation and investigation which must be definitely settled before there can be any talk of active work along the lines laid down at the International Congress. A very interesting subject for discussion at the meeting has been handed in by the Hungarian delegates—"In how far can the ordinary medical and post-graduate instruction in matters of hygiene in the several countries be considered as equivalent with a view to ultimate reciprocal permission to practise?" One of the probable results of the successful working of the Committee, we have no doubt, will be the closer union of medical men throughout the world, and the adoption of a system of reciprocity that is founded on real, and not on spurious and merely theoretical, grounds by the countries participating in the work of the Committee. We shall follow the deliberations of the Committee with great interest, and we sincerely hope that future meetings will see the post-graduate teaching associations of this country better represented on it than is at present the case. Dr. Pavy, we are informed, has been compelled, owing to want of time, to resign his membership of the Committee as well as of the Central Council, and in the circumstances, according to the statutes of the Committee, the British Government will be called upon to nominate his successor. There is a wide choice of eminent men who have shown that they have the cause of graduate study at heart in this country, but we think that no more universally popular and no more pre-eminently suitable selection can be made than that of Sir Jonathan Hutchinson to replace Dr. Pavy. If Sir Jonathan has the time and the inclination to serve on the Committee his counsel and advice will be of the greatest assistance.

### Barbarous School Punishments.

THERE is a great deal to be said for and against corporal punishment in schools, and the subject is obviously too important and too wide altogether to be dealt with in the limits of a paragraph. At the same time, no matter whether one is in favour of, or wholly opposed to, the rigid application of the Solomonian doctrines of juvenile education, there can be no question that the methods which appear to prevail in an Oxford College school, and which were recently made the subject of an inquiry in a police court, will have the whole-hearted condemnation of every right-thinking reader, be he medical man or lay. At this institute, it appears from the evidence, older boys are in the habit, ostensibly for the purpose of enforcing their authority, of striking younger pupils in the face. We are amazed to find such methods in an institute which is presumably more or less up to date in other respects, and where the pupils are, also presumably, healthy-minded English boys with some idea of the fitness of things. The blame of the case, we venture to think, rests entirely upon the head-master. No teacher in a public or private school can be ignorant of the methods of maintaining discipline which are used by prefects or older pupils, and where such methods are so eminently barbarous and (we say it deliberately) dangerous, the proper persons to be censured are those in authority. Medical men are fully aware of the lamentable consequences that often result from the pernicious habit of boxing children's ears or otherwise striking them on the head or in the face. It is, however, high time that laymen, and especially teachers, should be made acquainted with these results. We are by no means in unison with the sickly sentimentality that wishes to restrict the disciplinary powers of schoolmasters to mere methods of intellectual and moral, as opposed to physical, torture, but we say emphatically that the teacher who so far forgets himself as to strike a pupil in the face or on the head should be summarily indicted for common assault.

### Falsehood as Therapeutics.

LIKELY enough the "pass and fell incensèd points of mighty opposites" are just as hard to bring together nowadays as formerly. The difference, at any rate in matters medical, is that the current tendency is to bridge the gap with a network of ingenious phrases, whereas our fathers were not afraid to acknowledge the difficulties involved in the effort of crossing. We are, perhaps, more discriminating; they certainly gave much the stronger impression of intellectual honesty. In Robertson's early Victorian comedy, "Progress," now being revived at the Coronet Theatre, Dr. Brown, an uncompromising Radical and materialist, whose heroes are Cromwell and Jack Cade, has as patient the niece of an aged nobleman with strongly reactionary notions. This young lady is unfortunate enough to entertain affection for a young engineer employed by a railway company which wants to plant its station in the middle of her uncle's park. Her rather desperate state (the disease looks like pulmonary phthisis complicated with chlorosis) obtains for her an interview with her lover, and the physician assures her the course of true love is running quite smoothly. But poor Dr. Brown would never have done, supposing him extant at the present day, to have written an acceptable article on faith-healing, because he adds the highly successful aside, "What an infernal liar I am!" However, he makes the lady's relations back him up in his merciful deception, prompts their replies to her questions, and, finally overcoming all prejudices, forces on them the only prescription he will give, namely, "the engineer." No doubt his strength of purpose has a good deal to do with the fact that his line of treatment is adopted. It might be possible now to improve—though not very much—on worthy Dr. Brown's methods (as regards the ventilation of a sick-room, for instance), but, unfortunately, such downrightness and masterfulness do not seem to temper the profession in the present day.

